# Trans. Am. Med. Ass. 1856

**Published:** 1857-06

**Authors:** 


*Transactions of the American Medical Association for* 1856.—Prof. McGugin informs us that he has just received from Dr. Caspar Wistar, one dozen copies of the above volume, for sale to physicians or societies ; price $3 00. Prof. McGugin will transmit by mail or otherwise, to those sending him the above sum, and postage, when to be sent by mail. We doubt not they will find a ready sale, for a physician can hardly afford to be without so valuable a volume in his library.

